# Mechanical and Degradation Properties of Hybrid Scaffolds for Tissue Engineered Heart Valve (TEHV)

**DOI:** 10.3390/jfb12010020

**Published:** 2021-03-09

**Authors:** Rabia Nazir, Arne Bruyneel, Carolyn Carr, Jan Czernuszka

**Affiliations:** 1 Department of Materials, University of Oxford, Parks Road, Oxford OX1 3PH, UK; jan.czernuszka@materials.ox.ac.uk; 2 Interdisciplinary Research Centre in Biomedical Materials (IRCBM), COMSATS University Islamabad (CUI), Lahore Campus, Lahore 54000, Pakistan; 3 Department of Physiology, Anatomy and Genetics, University of Oxford, Parks Road, Oxford OX1 3PT, UK; arne.bruyneel@gmail.com (A.B.); carolyn.carr@dpag.ox.ac.uk (C.C.)

**Keywords:** compressive modulus, dynamic mechanical properties, enzymatic degradation, crosslinking density, aortic valve repair

## Abstract

In addition to biocompatibility, an ideal scaffold for the regeneration of valvular tissue should also replicate the natural heart valve extracellular matrix (ECM) in terms of biomechanical properties and structural stability. In our previous paper, we demonstrated the development of collagen type I and hyaluronic acid (HA)-based scaffolds with interlaced microstructure. Such hybrid scaffolds were found to be compatible with cardiosphere-derived cells (CDCs) to potentially regenerate the diseased aortic heart valve. This paper focused on the quantification of the effect of crosslinking density on the mechanical properties under dry and wet conditions as well as degradation resistance. Elastic moduli increased with increasing crosslinking densities, in the dry and wet state, for parent networks, whereas those of interlaced scaffolds were higher than either network alone. Compressive and storage moduli ranged from 35 ± 5 to 95 ± 5 kPa and 16 ± 2 kPa to 113 ± 6 kPa, respectively, in the dry state. Storage moduli, in the dry state, matched and exceeded those of human aortic valve leaflets (HAVL). Similarly, degradation resistance increased with increasing the crosslinking densities for collagen-only and HA-only scaffolds. Interlaced scaffolds showed partial degradation in the presence of either collagenase or hyaluronidase as compared to when exposed to both enzymes together. These results agree with our previous findings that interlaced scaffolds were composed of independent collagen and HA networks without crosslinking between them. Thus, collagen/HA interlaced scaffolds have the potential to fill in the niche for designing an ideal tissue engineered heart valve (TEHV).

## 1. Introduction

The ‘tissue engineering triad’ consists of scaffolds, cells, and growth factors [1,2,3]. Scaffolds are seeded with cells before implantation and can be considered as the structural backbone of the resulting TEHV [4]. Therefore, biomechanical properties of the scaffold play a crucial role in the post-implantation performance of the resulting TEHV. TEHV and target tissue should mechanically match so that TEHV can serve as a substitute for the target tissue until the development of new tissue is completed [5,6]. Scaffolds are designed to house the cells and give them sufficient support until a fully functional tissue can be generated [7,8,9]. Apart from mechanical strength, the second most important parameter for scaffold is the rate at which it degrades inside the body [10,11,12]. It should degrade at an appropriate rate into body-friendly by-products, which can be easily washed away by physiological fluids [13,14,15].

The heart valve is a dynamic tissue which requires an immediate fully functional TEHV [16]. The heart valve experiences different types of stresses such as flexure, bending, and tension during each cycle. Therefore, a scaffold aiming at the restoration of the traumatized tissue should be able to withstand 30 million such stress cycles a year [17,18,19]. Structurally, the cross-section of a leaflet of aortic valve is a tri-layered structure composed of fibrosa, spongiosa, and ventricularis responsible for the anisotropic mechanical behavior of the valvular tissue in the direction as well as across the layers [19,20]. Finite element analysis has shown that the principal load is supported in the circumferential direction during diastole and compressive forces are confined only to the boundaries [21,22]. Therefore, hybrid scaffolds in the present study could potentially be exploited for mimicking a load bearing layer, with the fibrosa as a preliminary model for TEHV, and have been studied under the effect of compressive and viscoelastic forces.

An 1-ethyl-3-(3-dimethylaminopropyl) carbodiimide hydrochloride (EDC) and *N*-hydroxysuccinimide(NHS)-based sequential crosslinking protocol, to create collagen/HA-based hybrid scaffolds with interlaced microstructure, were elucidated in our previous paper [23]. It is hypothesized that microscale interlacing between collagen type I and HA could affect the mechanical properties of the resulting scaffolds. Hence, this paper is based on the mechanical characterization of the parent networks (collagen type I and HA) and hybrid scaffolds. The compositions of hybrid scaffolds were selected so as to provide the combinations of least crosslinked (e.g., C0 and HA2) and highest crosslinked networks (e.g., C60 and HA20) in order to quantify the variation of various properties over the range of crosslinking density spectrum. Scaffolds have been characterized under simple compression and dynamic modes. The TEHV will come in contact with physiological fluids, e.g., cell culture medium and/or blood which could affect its mechanical properties [24,25]. Therefore, dynamic properties under wet conditions have also been performed in this study. Degradation behaviors of biocompatible polymers, either synthetic or natural, depend on various factors such as physical (crystallinity and molecular weight) and chemical properties (nature of bonding) of the polymer [26,27,28]. The degradation rate and degradation mechanism are also affected by the host environment [29]. For example, the in vivo degradation rate is usually higher than in vitro degradation rate. Degradation in non-enzymatic physiological fluids (in vitro) occurs through hydrolysis whereas polymer chain cleavage in the presence of enzymes further accelerates the degradation under in vivo conditions [30,31]. The degradation behavior is usually studied in phosphate buffer saline (PBS, pH 7.4) at 37 °C with or without enzymes [32,33]. In this piece of work, the degradation behavior of parent networks and hybrid scaffolds with and without enzymes under the standard condition was investigated. It is also important to elucidate the degradation behavior of scaffolds under different pH, where pH 7.4 represents physiological environment and pH 4.0 represents the environment of an infected tissue of body [34,35]. The non-enzymatic experiments were replicated under acidic and basic conditions to understand the degradation behavior on a broader spectrum. Furthermore, the effect of variation in crosslinking density on degradation resistance of parent networks and hybrid scaffolds was also discussed. The behavior of hybrid scaffolds in the presence of enzymes (collagenase and hyaluronidase) was examined such that collagen/HA hybrid scaffolds were exposed to each enzyme individually and together. It is hypothesized that collagenase could attack the collagen network only without affecting the HA network and vice versa. 

## 2. Materials and Methods

### 2.1. Synthesis and Crosslinking of Parents and Hybrid Scaffolds

**Figure 1:**
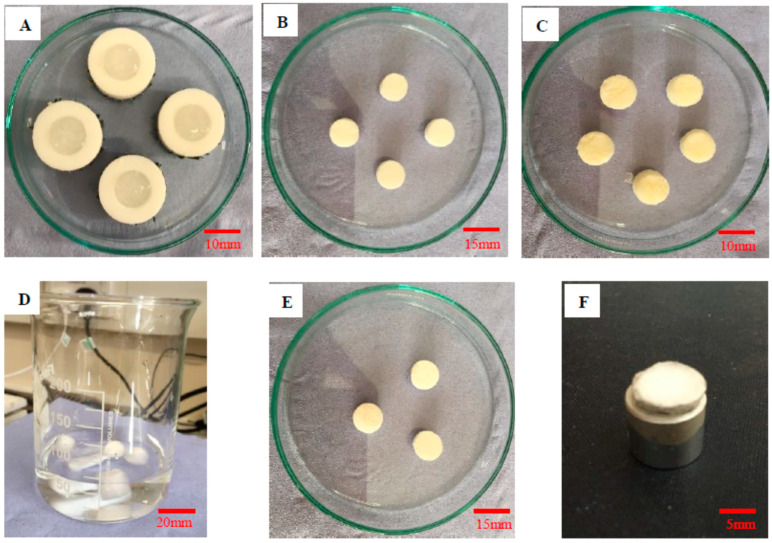
(**A**) Collagen suspension in PTFE molds, (**B**) freeze-dried collagen scaffolds, (**C**) freeze-dried HA scaffolds, (**D**) Collagen scaffolds in crosslinking HA solution, (**E**) freeze-dried collagen/HA hybrid scaffolds, and (**F**) platinum-coated collagen/HA scaffold sample for SEM.

All chemicals were purchased from Sigma Aldrich, UK unless stated otherwise. Briefly, primary collagen scaffolds were prepared by making 1% collagen type I suspension in 0.05 M acetic acid (pH 3.2) using a kitchen blender, frozen overnight at −20 °C, and freeze-dried for 24 h. Half of the scaffolds were left uncrosslinked (C0) at this stage. The remaining scaffolds were cross-linked by 60 mM EDC/30 mM NHS for 4 h followed by washing in 1 M sodium chloride (NaCl) solution. Finally, scaffolds (C60) were frozen at −20 °C overnight followed by freeze-drying. For secondary HA scaffolds, sodium hyaluronate (SHA, ACROS organics) solution was prepared at 4 mg/mL followed by addition of 30 time molar excess of adipic dihydrazide (ADH). Different amounts of EDC were added to the above solution to prepare (2 mM, 4 mM, 10 mM, 20 mM) while maintaining the pH at 4.75, stirred for 24 h, and dialyzed twice against deionized water for another 24 h before freeze drying. Collagen/HA hybrid scaffolds were prepared by combining the above two networks such that un-crosslinked and crosslinked collagen scaffolds (i.e., C0 and C60) prepared described above were placed in each of the dialyzed HA solutions (2 mM and 20 mM) for 24 h with gentle stirring. HA formed the secondary network through the primary collagen network. The resulting hybrid scaffolds were removed from the HA reaction mixture, washed, and freeze dried as described above [20]. Synthesis steps are shown in Figure 1 and the nomenclature of parent as well as hybrid compositions is given below in Table 1 and Table 2. 

### 2.2. Characterizations

#### 2.2.1. Compressive Test

Unidirectional compression tests, with a 50 N load cell, were conducted using a DMA7 (PerkinElmer, Waltham, MA, USA). Prior to freeze drying, top and bottom surfaces of all frozen scaffolds were levelled using a sharp razor blade in order to make a uniform complete with loading plates. Cylindrical samples (d = 15 mm, h = 15 mm), preloaded to 1 mN, were compressed between parallel plates at a constant strain rate of 0.5 mm/min. All samples (n = 5) were tested at room temperature in the dry state. Compression testing could not be performed under wet conditions due to the sensitivity limit of the testing equipment.

#### 2.2.2. Dynamic Mechanical Properties

Dynamic mechanical properties of collagen network and the resultant collagen/HA hybrid scaffolds (n = 5), under dry and wet conditions, were measured using a DMA Q800 (PerkinElmer, Waltham, MA, USA). Cylindrical samples (d = 15 mm, h = 15 mm) were subjected to cyclic forces (0 and 10% compressive strain) in the frequency range 0.01–100 Hz at 37 °C. For wet testing, samples were tested under same conditions while immersed in PBS bath.

#### 2.2.3. Rheology Test

Like collagen and hybrid scaffolds, the effect of increase in crosslinking density on the mechanical properties of HA network could be informative. However, compression and viscoelastic tests could not be conducted on HA networks due to the sensitivity limit of the testing equipment and fragile nature of the samples. Study of the rheological properties of HA networks could reveal the effect of crosslinking on their viscoelastic properties. Samples were characterized with an Anton Paar Physica MCR 301 compact rheometer using oscillatory shear stress test conditions. The tests were performed using parallel plate geometry (25 mm) with a gap size of 1 mm. Constant values of deformation, 0.1–1 mrad, were maintained throughout each frequency sweep of 1–10 Hz. Four samples from each group were soaked in PBS for 1 h before the test. 

#### 2.2.4. Degradation Behavior under Non-Enzymatic Conditions

Collagen scaffolds, HA scaffolds, and collagen/HA hybrid scaffolds (d = 12 mm, h = 4 mm) were prepared. Initial mass (M_1_) of each scaffold was recorded and the sample was incubated in 0.01M PBS (Sigma-Aldrich, Gillingham, UK, pH 7.4) at 37 °C. Three scaffolds from each group were removed on day 1, 7, 14, 21, and 28. The samples were washed three times with deionized water to remove residual salts. These were then frozen at −20 °C overnight, freeze-dried for 24 h, and final mass (M_2_) was recorded. Mass loss in scaffolds at each time point as a result of degradation was calculated using the following equation:

Mass Loss = [(M_2_ − M_1_)/M_1_] × 100 (1)

The study was repeated in PBS at pH 4.0 and pH 10.0 to study the effect of pH on the degradation behavior.

#### 2.2.5. Degradation Behavior under Enzymatic Conditions

Collagen/HA hybrid scaffolds (d = 12 mm, h = 4 mm) were exposed to 0.01 M PBS (pH 7.4) containing collagenase from *Clostridium histolyticum* (≥250 CDU/mg of solid, Sigma-Aldrich, UK) and hyaluronidase from *streptomyces hyalurolyticus* (400–1000 CDU/mg of solid) at a concentration of 10 U/mL of each at 37 °C for 35 h. Three scaffolds from each composition were removed after 1, 5, 8, 24, and 35 h. The samples were washed three times with deionized water to remove residual salts, frozen overnight at −20 °C, and freeze-dried for 24 h. Final mass (M_2_) was recorded and percentage mass loss was calculated using Equation (1). The experiment was repeated with the only collagenase and also with only hyaluronidase. The parent networks, e.g., collagen (C0 and C60) and HA (HA2 and HA20), were degraded in collagenase and hyaluronidase, respectively as controls under the same conditions (10 U/mL of each enzyme, 37 °C, 35 h). 

#### 2.2.6. Statistical Analysis

All data were reported as means of at least four samples, with the standard deviations as the errors. One-way analysis of variance (ANOVA) with a Bonferroni’s post hoc test was used to compare groups of samples with a probability value of 95 % (*p* < 0.05) to determine statistical significance. 

## 3. Results

### 3.1. Crosslinking Density Calculations

The crosslinking densities of parent as well as hybrid scaffolds were calculated from swelling studies data, as reported in our previous paper, using the following version of the Flory–Rehner equation, and are given below in Table 3 [23].

v_e_ = ρ_p_/M_c_ (kmol/m^3^) (2)

where 

ρ_p_ = Density of the dry polymer (1.229 gm/cm^3^ for HA, 1.35 gm/cm^3^ for collagen)

M_c_= Average molecular weight between the two crosslinks (kg/mol).

### 3.2. Compression Test

The compression moduli (E*_comp_*, kPa) for all samples are given in Table 4.

Table 4 shows that E*_comp_* for collagen networks increases from 35 ± 5 to 68 ± 7 kPa with an increase in v_e_. Similarly, E*_comp_* for hybrid scaffolds increases from 41 ± 4 to 95 ± 4 kPa. Comparison shows that *E_comp_* values of hybrid compositions are 1.4–2.0 times higher than those of parent collagen networks. *E_comp_* of hybrid compositions is also affected by the crosslinking density of the secondary HA network as can be seen from Table 4. For instance, HA20 (v_e_, 17 ± 4 kmol/m^3^) is the parent HA network in S-2 (*E_comp_*, 73 ± 5 kPa) and S-4 (*E_comp_*, 95 ± 4 kPa) and these compositions show higher *E_comp_* values as compared to S-1 (*E_comp_*, 41 ± 4 kPa) and S-3 (*E_comp_*, 68 ± 5 kPa) having HA2 (v_e_, 2 ± 0.3 kmol/m^3^) as parent HA network. 

### 3.3. Dynamic Mechanical Properties

**Figure 2:**
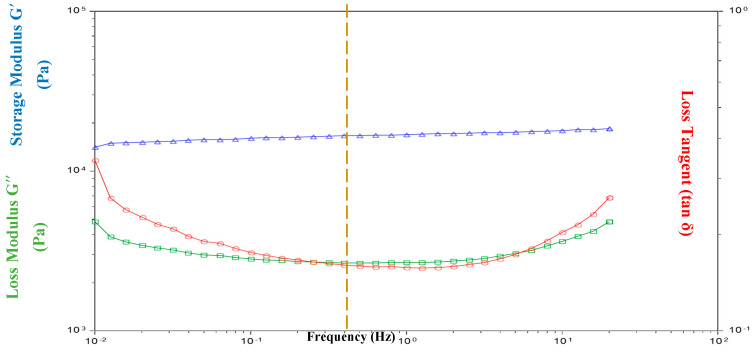
Variation of storage modulus G′ (Pa), loss modulus G″ (Pa) and loss tangent (tan δ) of the collagen network in the dry state at 37 °C. G′ > G″ suggests that collagen scaffold behaves as elastic solid. The dashed line indicates the reference point for the values of G′, G″ and tan δ as presented in Table 2 and Table 3.

Figure 2 shows the representative frequency sweep test for non-crosslinked collagen in the dry state at 37 °C. Similar tests have been run for collagen networks as well as the resultant hybrid scaffolds. The storage modulus, G′, represents the elastic character whereas loss modulus, G″, accounts for the viscous behavior of the material under given oscillatory conditions. Loss tangent, tan δ, shows material’s internal friction and is defined as is the ratio of G″/G′. Figure 2 suggests that G′ is independent of the frequency, f. At low frequencies, G″ and tanδ are nearly constant, whereas they tend to increase slightly towards the end of the test. Overall, all parameters show a monotonous behavior in the range f = 10^−1^–0^1^. The frequency of a healthy adult human heart (assuming 70 beats per minute) is 1 Hz [36]. Therefore, taking it as a reference point, the values of G′, G″, and tan δ for collagen networks and hybrid scaffolds are given in Table 5 and Table 6 for dry and wet conditions, respectively. 

Table 5 shows that the dynamic moduli, G′ and G″, increase with increasing the crosslinking density for collagen networks and hybrid scaffolds. G′> G″ and tanδ < 1 for all samples. There is a five-fold increase in G′ and a three-fold increase in G″ from C0 to C60. G′ and G″ also increase steadily from S-1 to S-4. For instance, S-4 (G′, 113 ± 6 kPa) is almost two times more elastic than S-1(G′, 52 ± 6 kPa) under given conditions. Comparison shows that the dynamic moduli of hybrid scaffolds are two to four times higher than their collagen networks. For example, S-4 (G′, 113 ± 6 kPa) is more elastic than its parent collagen network C60 (G′, 74 ± 3 kPa). Tanδ for collagen networks, as well as for hybrid scaffolds, decreases, from 0.17 to 0.12 and from 0.12 to 0.09, respectively, with increasing the crosslinking density as shown in Table 2. It also indicated that tanδ for hybrid scaffolds is lower than for their parent collagen networks.

Table 6 shows that G″> G′ and tanδ > 1 for sample C0; however, this equilibrium shifts with crosslinking as G′ > G″ and tanδ < 1 for all other samples. Comparison of Table 5 and Table 6 indicates that G″ and G′ for all samples except C0 decrease in the hydrated state 2–7 times as compared to those in the dry state. Tanδ increases for all samples in the hydrated state, as shown in Table 6. It also shows that tanδ has lower values for all hybrid scaffolds as compared to collagen networks. Similar to the dry test, scaffolds with higher crosslinking densities have higher G′ and G″ in wet test or vice versa. For instance, comparison shows that G′ and G″ are higher for hybrid scaffolds as compared to their parent collagen networks. The degree of crosslinking of collagen network also affected tanδ in wet condition. For instance, S-1(tanδ, 0.38) and S-2 (tanδ, 0.14), with C0 (tanδ, 1.17) as parent network, have higher values as compared to S-3 (tanδ, 0.1) and S-4 (tanδ, 0.1) which have C60 (tanδ, 0.18) as parent network. 

### 3.4. Rheology Testing 

Table 7 shows the effect of an increase in crosslinking density on rheological properties of the HA network. G′ increases with increasing the crosslinking density from 320 ± 24 Pa for HA2 to 870 ± 21 Pa for HA20. Similarly, G″ also increases from 19 ± 6 Pa for HA2 to 33 ± 4 Pa for HA20 with increasing the crosslinking density. As a result, tanδ decreases from 0.05 to 0.03 (40 % reduction). Comparison indicates that G′ and G″ for collagen networks are up to 20 times higher than HA networks. In addition, the range of tanδ for collagen networks (1.17–0.18) is up to 25 times higher than that of HA networks (0.05–0.03) in wet condition. 

### 3.5. Degradation Behavior under Non-Enzymatic Conditions

The degradation behavior of parent and hybrid scaffolds under the effect of different pH values (PBS, 37 °C, 28 days), are presented in the following sections.

#### 3.5.1. Collagen Scaffolds

**Figure 3:**
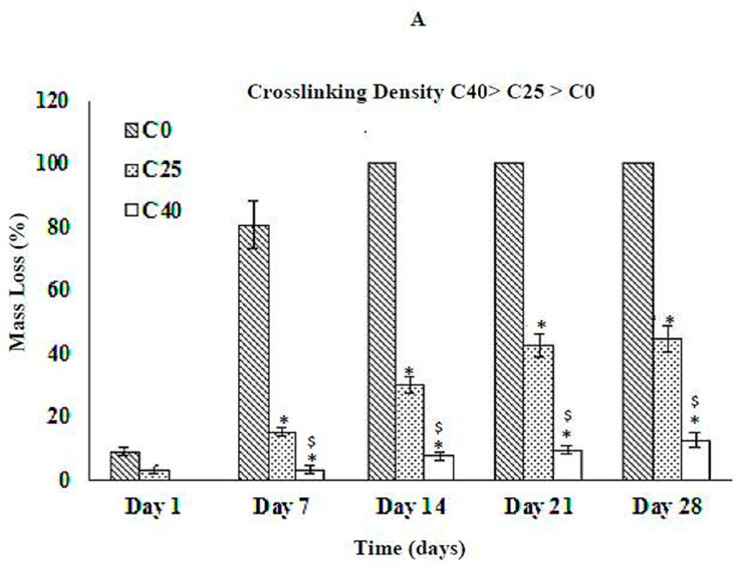

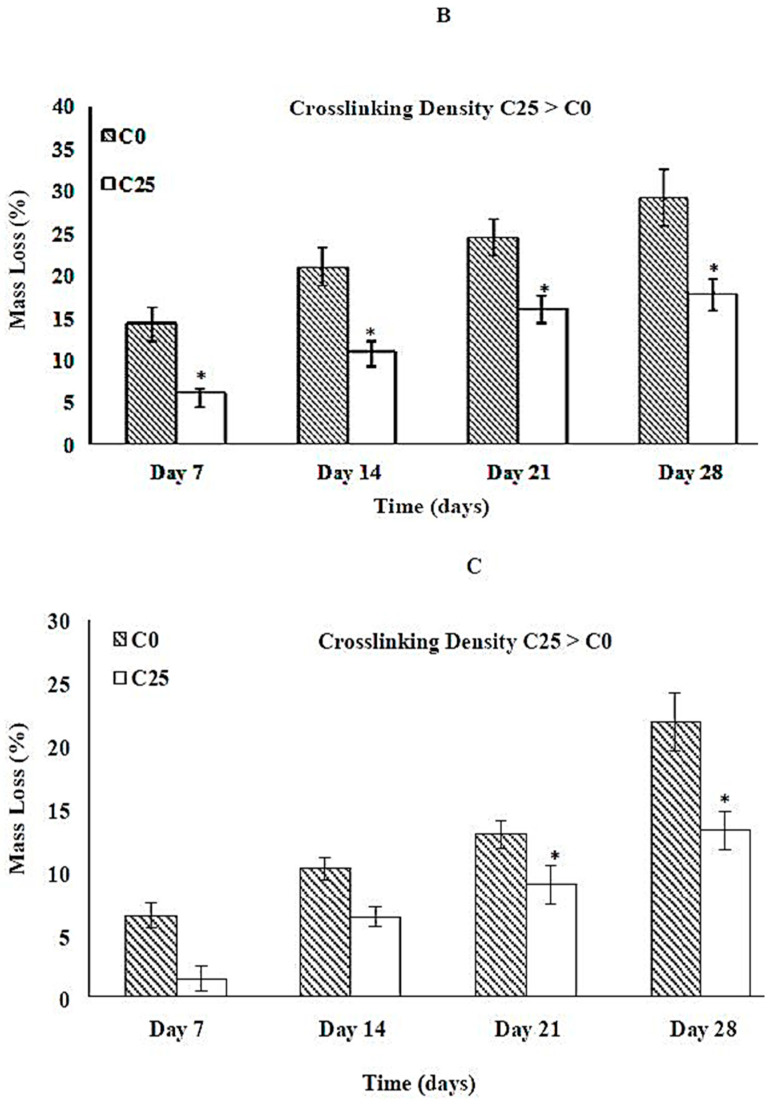
Effect of crosslinking density on the degradation behavior of collagen scaffolds at (**A**) pH 4.0, (**B**) pH 7.4, and (**C**) pH 10. * and ^$^ represent statistically significant values (*p* < 0.05) within collagen scaffolds on a given day as compared to C0 and C25, respectively.

The increase in crosslinking density increases the degradation resistance of collagen network at pH 4.0, 7.4, and 10.0 as shown in Figure 3A, Figure 3B, and Figure 3C, respectively. Mass loss decreases as the crosslinking density increases at all three pH values. There is no mass loss for any composition at pH 7.4 and 10 on day 1 (Figure 3B,C). The mass loss for sample C40 is almost negligible at pH 7.4 and 10.0. Similarly, C60 do not show any mass loss after 28 days at pH 4.0, 7.4 and 10.0. Comparing Figure 3A–C, mass loss is highest for acidic pH and lowest for basic pH, which implies that the order of degradation for collagen networks is as follows:

Acidic > Neutral > Basic

#### 3.5.2. HA Scaffolds

**Figure 4:**
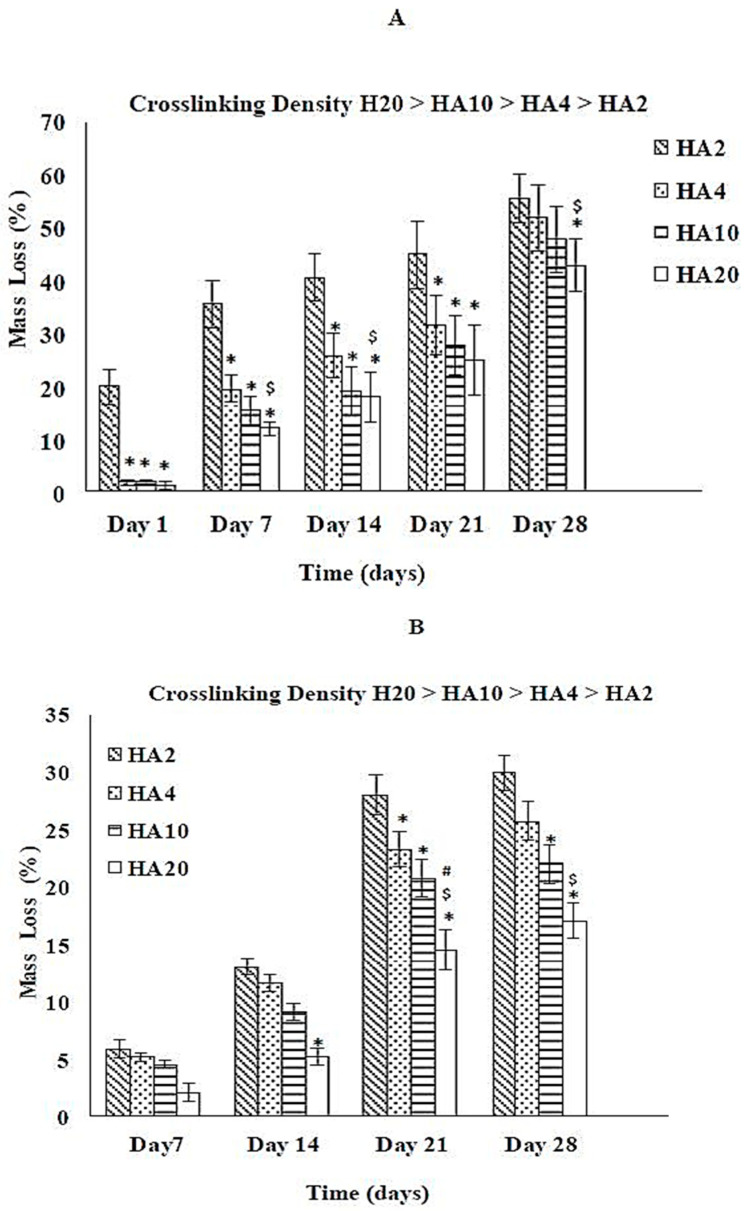
Effect of crosslinking density on the degradation behavior of HA scaffolds (**A**) pH 4.0 and (**B**) pH 7.4. *, ^$^ and # represent statistically significant values (*p* < 0.05) within HA scaffolds on a given day as compared to HA2, HA4, and HA10, respectively.

Similar to the collagen scaffolds, an increase in the crosslinking density increases the degradation resistance of HA scaffolds under acidic and physiological conditions as shown in Figure 4A,B. HA networks exposed to pH 10.0 could not survive beyond day 1 (data not shown). As the EDC concentration is increased from 2 to 20 mM, the crosslinking density also increases and the total mass loss decreases from 30% to 17% and 55% to 43% at pH 4.0 and pH 7.4, respectively. There is no mass loss for any composition on day 1 at pH 7.4 (Figure 4B). Comparing Figure 4A and Figure 4B, mass loss is highest for basic pH and lowest for acidic pH, which implies that the order of degradation for HA networks is as follows:

Basic > Neutral > Acidic

#### 3.5.3. Collagen/HA Hybrid Scaffolds

**Figure 5:**
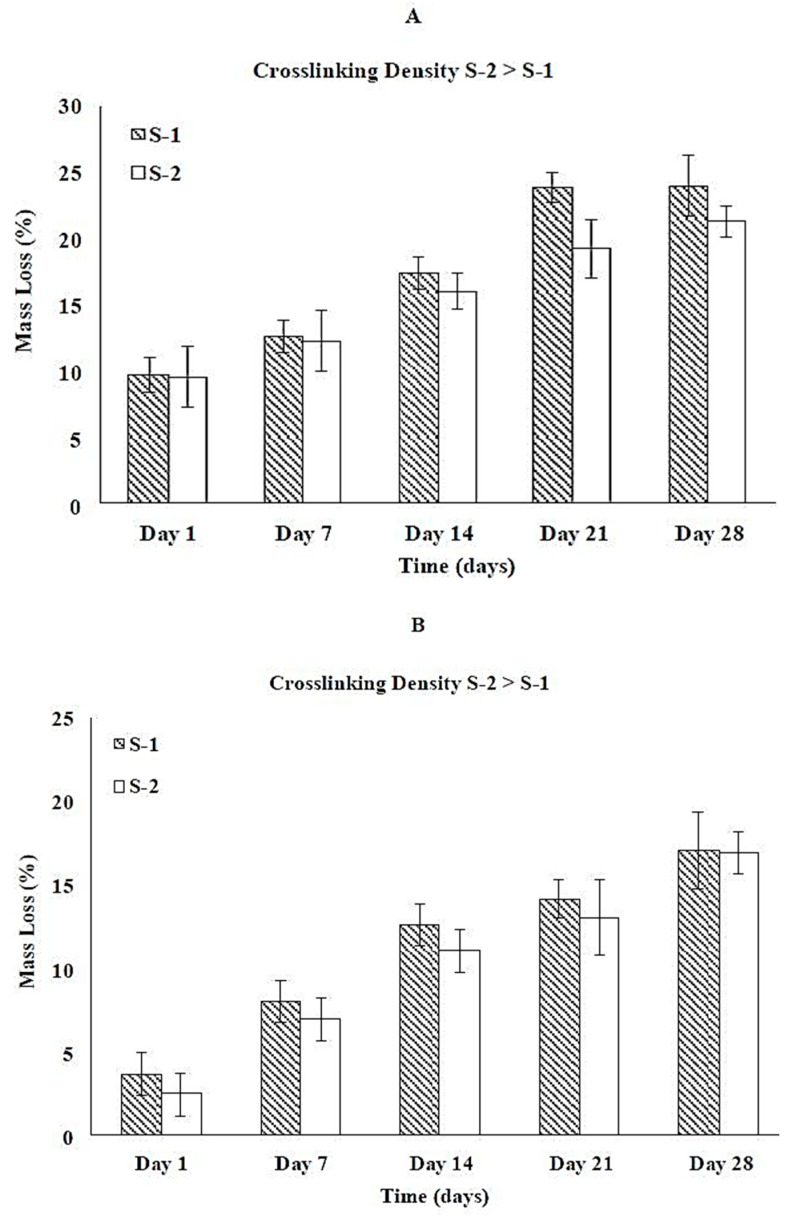

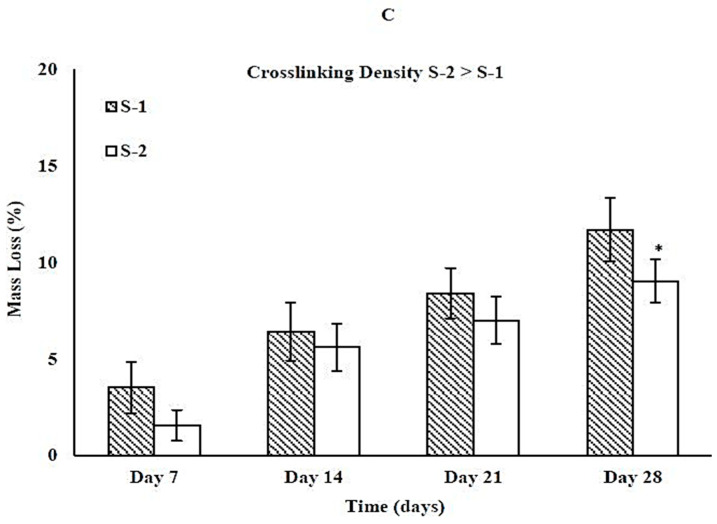
Effect of crosslinking density on the degradation behavior of collagen/HA hybrid scaffolds at (**A**) pH 4.0, (**B**) pH 7.4, and (**C**) pH 10.0. S-3 and S-4 did not show any mass loss. * represent statistically significant values (*p* < 0.05) within collagen/HA hybrid scaffolds on a given day as compared to S-1.

Degradation behavior of collagen/HA hybrid scaffolds at pH 4.0, 7.4, and 10.0 is shown in Figure 5A–C, respectively. While comparing the total mass loss of S-1 and S-2 under different pH as shown in Figure 5A–C, mass loss is highest at pH 4.0 and lowest pH 10.0. There is no mass loss for any composition on day 1 at pH 10.0 (Figure 5C). S-3 and S-4 do not show any mass loss until 28 days under all conditions. Figure 5A–C suggests that the order of degradation for collagen/HA hybrid scaffolds under non-enzymatic conditions is as follows:

Acidic > Neutral > Basic

The total mass loss for collagen/HA hybrid scaffolds is less than the total mass loss for either collagen scaffold or HA scaffold. There is no significant difference in the mass loss between S-1 and S-2 under all conditions. 

### 3.6. Degradation Behavior under Enzymatic Conditions

**Figure 6:**
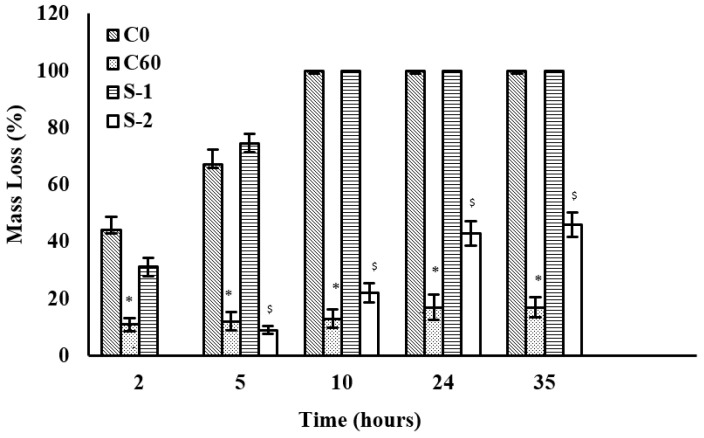
Degradation behavior of collagen/HA-based hybrid scaffolds in the presence of collagenase (10 U/mL, PBS pH 7.4, 35 h). Collagen networks (C0 and C60) were degraded by respective enzymes as controls. * and ^$^ represent the statistically significant values (*p* < 0.05) as compared to C0 and S-1, respectively within controls and hybrid scaffolds.

Degradation behavior in the presence of collagenase at 37 °C (pH 7.4, 10 U/mL) until 35 h is shown in Figure 6. C0 and C60, as controls, seem to span the range of the degradation behavior of the hybrid scaffolds. C0 completely degrades in 10 h but the mass loss for C60 is only 17% after 35 h. S-1 shows degradation behavior similar to C0. S-2 shows a total mass loss of 46% in 35 h. 

**Figure 7:**
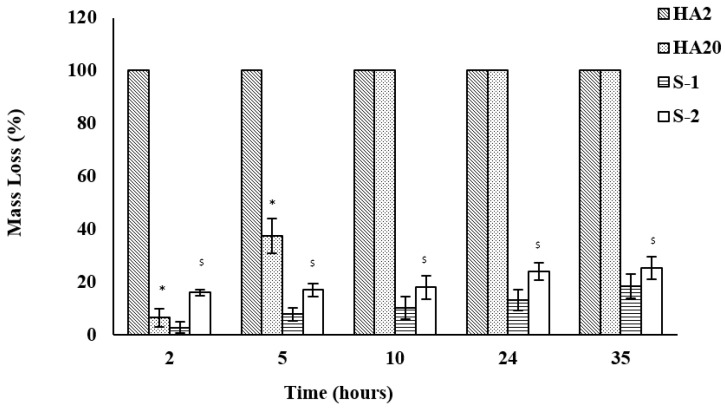
Degradation behavior of collagen/HA hybrid scaffolds in the presence of hyaluronidase (10 U/mL, PBS pH 7.4, 35 h). HA networks (HA2 and HA20) were degraded by respective enzymes as controls. * and ^$^ represent the statistically significant values (*p* < 0.05) as compared to HA2 and S-1, respectively within controls and hybrid scaffolds.

Hybrid scaffolds are degraded in the presence of hyaluronidase (10 U/mL, pH 7.4, 35 h). HA2 and HA20 are run as controls and exhibit low degradation resistance as shown in Figure 7. HA2 survives for 2 h, whereas HA20 degrades completely in 10 h. S-1 and S-2 show total mass loss of around 19% and 25%, respectively. 

**Figure 8:**
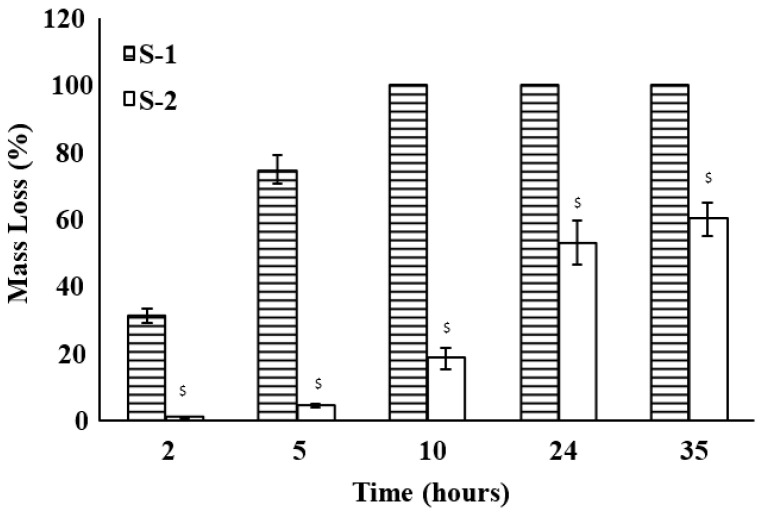
Degradation behavior of collagen/HA hybrid scaffolds in the presence of collagenase and hyaluronidase (10 U/mL of each, PBS, pH 7.4) at 37 °C for 35 h. ^$^ represents the statistically significant values (*p* < 0.05) as compared to S-1. S-3 and S-4 did not show any mass loss under given conditions.

Degradation behavior of hybrid scaffolds under the combined effect of collagenase and hyaluronidase (10 U/mL of each, pH 7.4, 35 h) is shown in Figure 8. The degradation rate is higher as compared to degradation under the effect of collagenase or hyaluronidase alone, as shown in Figure 6 and Figure 7. S-1 is digested completely in 10 h, which is similar to the behavior under the effect of collagenase only (Figure 6). S-2 has a relatively higher degradation resistance and shows a total mass loss of 60% in 35 h. Collagenase and hyaluronidase alone or together have no effect on S-3 and S-4 and hence no appreciable mass loss could be measured for them. Taking Figure 6, Figure 7 and Figure 8, the order of degradation rate of collagen/HA hybrid scaffolds in the presence of enzymes can be defined as:

Collagenase + Hyaluronidase > Collagenase > Hyaluronidase

## 4. Discussion

It was shown in our previous paper that increase in the concentration of crosslinking agent (i.e., EDC/NHS) resulted in an increase in the crosslinking density of collagen networks, HA networks, and hybrid scaffolds which in turn affected the cardiosphere derived cell (CDCs) response [23]. The effects of these factors on the mechanical and degradation properties will be discussed in the following sections.

### 4.1. Effect of Crosslinking Density and Interlacing on E_comp_

Increase in E*_comp_* of collagen networks with increasing the crosslinking density can be explained by considering the deformation of a single pore within an elastomeric foam (or scaffold). An elastomeric foam deforms by the pore edge bending and pore wall buckling under the effect of compressive stress. It was shown in our previous work that the pore size of collagen networks tends to decrease when increasing the crosslinking density, possibly due to the volume shrinkage (Δv) [23]. A reduction in pore size could increase the resistance towards pore edge deformation and pore wall buckling which would increase the E*_comp_* of collagen networks [37,38,39]. Table 4 shows that E*_comp_* values of hybrid scaffolds are higher than their parent collagen network. It could be due to the effect of interlocking between collagen and HA networks (see Figure S1, supplementary materials). Table 4 also suggested that incorporation of HA network with higher crosslinking density (HA20) increased the E*_comp_* up to 2 times as compared to the one with lower crosslinking density (HA2). The higher crosslinking density of HA20 could result in higher degree of interlocking with collagen network in S-2 and S-4 which could result in higher E*_comp_* for these compositions. 

### 4.2. Effect of Crosslinking Density and Interlacing on Dynamic Mechanical Properties

Dynamic mechanical properties give an insight into microstructural changes resulting from polymer chain movements [40,41]. Table 5 shows that the dynamic moduli (G′, G″) of collagen network increase with increasing the crosslinking density. This could be due to the fact that zero-length crosslinks, formed by EDC/NHS, act as joining points between polymer chains resisting chain slippage [42,43]. This increased resistance, due to higher crosslinking density, against slipping under the effect of dynamic forces could explain the increase in G′ and G″ for primary collagen networks. Table 5 shows that values of dynamic moduli for hybrid compositions are greater than collagen networks. Increase in G′ and G″ for hybrid scaffolds could be due to the incorporation of secondary HA network which is likely to further restrict the polymer chains. tanδ < 1 shows that elastic character is dominant for all scaffolds in the dry state and decrease in tanδ with increasing the crosslinking density indicates an increase in the elastic character. S-3 and S-4 have lowest tanδ suggesting that polymer chain movement is the most restricted in these compositions due to polymer chain entanglements between collagen type I and HA.

### 4.3. Effect of Crosslinking Density on the Mechanical Properties under Wet Conditions

Comparison of Table 5 and Table 6 suggests that G′ and G″ for collagen networks decrease significantly in the wet state as compared to those in the dry state. Our previous study has shown that –COOH and –NH_2_ groups form the amide bond during the EDC/NHS crosslinking [23]. The decrease in water uptake is possibly because of the engagement of these water-interacting sites (–COOH, –NH_2_) in the crosslinking process which could increase the hydrophobicity of collagen network [44]. Increased hydrophobicity could result in taking up less water by the scaffolds with higher crosslinking densities such as C40 and C60. Overall, water taken up by the scaffolds may act as a lubricant to facilitate chain slipping which explains the decrease in G′ and G″ for collagen networks in wet condition. Comparison of tanδ values from Table 5 and Table 6 also supports this explanation that collagen chains experience more freedom in the wet state as compared to dry state most likely due to the lubricating effect of the absorbed water.

In addition to the effect of crosslinking density, water intake of hybrid compositions could also be related to the presence of secondary HA network [45,46,47]. In the hydrated state, HA chains could slip off easily out of the collagen network and the role of primary collagen network becomes important at this stage. The presence of highly crosslinked collagen network (C60) in hybrid compositions (S-3 and S-4) could hold back the hydrated HA chains more efficiently than non-crosslinked collagen network (C0) present in S-1 and S-2. This suggests that hydrophilic effect of HA chains probably dominates the hydrophobic effect of collagen network for hybrid scaffolds in the wet state.

According to Table 6, the shift from the viscous character for C0 (G″ > G′ and tanδ > 1) to the elastic character (G″ < G′ and tanδ < 1) for all other compositions suggests that crosslinking contributes towards increasing the elastic character of the scaffolds. Comparison of Table 5 and Table 6 shows that G′ values for hybrid scaffolds are higher than those of collagen networks whereas there is no significant difference between G″ values for collagen networks and hybrid scaffolds. Tanδ values for S-1 and S-2 that presence of HA chains tend to experience more freedom due to the lack of hold from non-crosslinked C0 network. On the other hand, tanδ decreases for S-3 and S-4 where highly crosslinked C60 network could hold back the HA chains from slipping. 

### 4.4. Comparison of Mechanical Properties of Hybrid Scaffolds with the Human Aortic Valve Leaflet (HAVL)

Many research groups have been investigating the mechanical properties of the heart valve leaflets [48,49]; however, the available data are highly divergent due to the biological diversity and lack of standardization in testing methods [50,51]. Therefore, the comparisons of mechanical properties from existing literature, presented here, represent only close approximation to TEHVs. Table 4, Table 5, Table 6 and Table 7 indicate that hybrid scaffolds showed higher mechanical properties as compared to the either network alone. E*_comp_* of HAVL has not been reported yet whereas that of the porcine aortic valve leaflet (PAVL) from belly region has been reported in the range of 5–6 kPa [52]. E*_comp_* of hybrid scaffolds is in the range of 41–95 kPa; which exceeds that of PAVL. Dynamic moduli (dry state) of the collagen/HA hybrid scaffolds, as reported in this paper, have been compared with those of HAVL in Table 8. G′ values of hybrid scaffolds are 5–10 times higher than those of HAVL and sinus. Additionally, tanδ for HAVL is higher than those of hybrid scaffolds. This comparison suggests that hybrid scaffolds, in a dry state, are stiffer and more elastic than HAVL.

Lower G′ and higher tanδ of HAVL, from Table 8, could be due to the hydrated condition of the tissue and therefore, Table 9 compares the wet mechanical properties of hybrid scaffolds with those of HAVL. Table 9 shows that G′ and tanδ of S-1 and S-2, in the wet state, match those of HAVL whereas S-3 and S-4 have higher values than those of HAVL. Dynamic mechanical properties of the hybrid scaffolds, in the wet state, match those of HAVL whereas scaffolds, in a dry state, seem more elastic than HAVL. This indicates that hybrid scaffolds could also be optimized for the target tissues stiffer than HAVL such as cartilage.

### 4.5. Effect of Crosslinking Density and Interlacing on the Degradation Properties under Non-Enzymatic Conditions

EDC/NHS crosslinking has increased the degradation resistance of the parent scaffolds when exposed to acidic, basic, and physiological environments probably because of the engagement of the water interacting sites (–COOH, –NH_2_) in the crosslinking process which could have increased the hydrophobicity of these polymers as discussed in our previous work [23,54]. Increase in hydrophobicity could slow down the degradation rate and hence less mass loss due to degradation [44,55]. In the case of hybrid scaffolds, S-1 and S-2 showed similar degradation rate whereas S-3 and S-4 did not show any mass loss under non-enzymatic conditions. This behavior can be attributed to the poor binding of non-crosslinked C0 network for HA network in S-1 and S-2 whereas the presence of highly crosslinked C60 network could hold the HA network in S-3 and S-4. 

In this study, the pH dependence of the degradation behavior of the parent networks and hybrid scaffolds was also investigated. For instance, the rate of hydrolysis of the peptide (or amide) bond of collagen is higher at acidic pH [56,57] and this could be the reason for the greatest mass loss of collagen scaffolds at pH 4.0 (Figure 3A). On the other hand, HA is known to degrade through the deacetylation of the N-acetyl group at basic medium which could result in rapid degradation rates [58,59]. This explains the greatest mass loss of the HA scaffolds at pH 10, as shown in Table 8. Comparison of Figure 3 and Figure 4 suggests that the pH dependence of the degradation behavior of the hybrid scaffolds follows that of the collagen scaffolds. This implies that the collagen network is abundantly present in the hybrid compositions and dominates the degradation behavior of these scaffolds (Figure 5). Furthermore, comparison of Figure 3 and Figure 4 shows that collagen network and HA network show contrasting behavior with respect to pH of the degrading medium. It can be inferred that pH-sensitive degradation of hybrid scaffolds can be tailored by choosing the primary network during fabrication.

### 4.6. Effect of Crosslinking Density and Interlacing on the Degradation Properties under Enzymatic Conditions

Enzyme-assisted degradation studies of collagen/HA hybrid scaffolds have been informative for understanding the in vitro degradation behavior of these scaffolds. Collagenase and hyaluronidase have been chosen to target the collagen and HA networks, respectively. When exposed to collagenase, the presence of HA network did not enhance the degradation resistance of S-1 and S-2 significantly (Figure 6). S-3 and S-4 showed high degradation resistance to collagenase which is most likely because of their higher crosslinking densities. Mass loss in all samples, in the presence of collagenase, could be attributed to the degradation of collagen network only. HA controls degraded quickly in the presence of hyaluronidase (Figure 7). This may be partly due to the bulk erosion arising from the highly hydrophilic character of HA [60] and partly due to the presence of hyaluronidase [61]. As the collagen is added to the HA network, degradation resistance towards hyaluronidase increases considerably as seen for S-1 and S-2. Mass loss of hybrid scaffolds in the presence of collagenase and hyaluronidase together is higher than the mass loss in the presence of collagenase or hyaluronidase alone (Figure 8). This implies that the mass loss resulted from the degradation of both collagen and HA networks in hybrid scaffolds. S-3 and S-4 maintained their non-degradability under the given conditions due to the extensive entanglements between collagen and HA networks, as shown in the SEM and histology images reported in our previous work [23,54].

**Figure 9:**
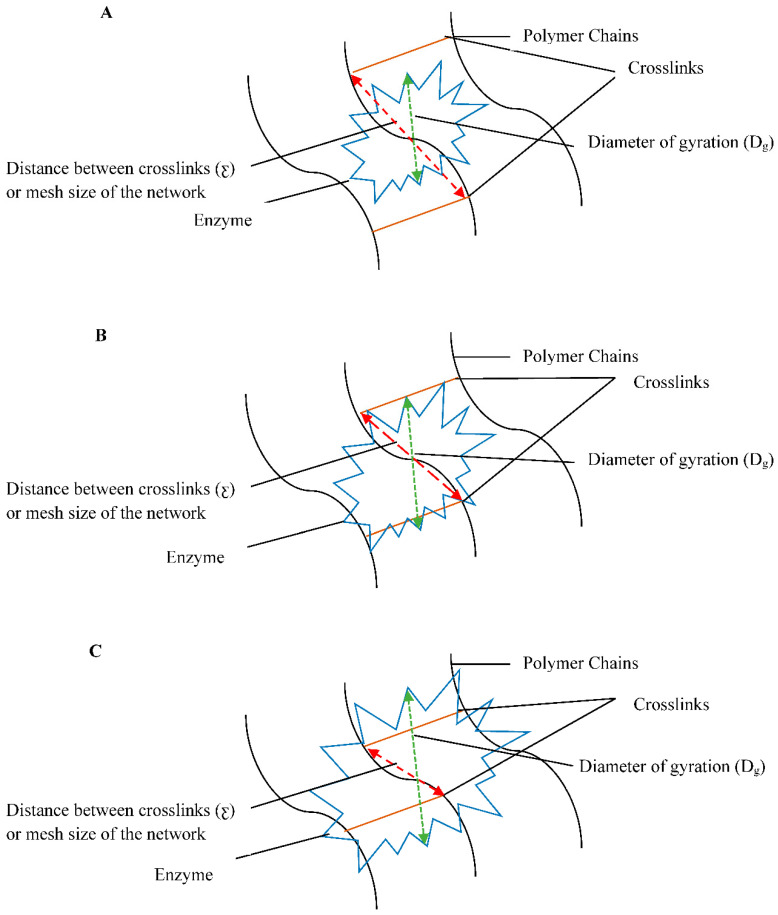
Schematic diagram for enzyme-assisted degradation of the crosslinked network (**A**) ƹ > D_g_, the degradation rate is high (**B**) ƹ ~ D_g_, the degradation rate is suppressed (**C**) ƹ < D_g_, degradation is stopped.

The mechanism of enzyme-assisted degradation can be understood by comparing the diameter of gyration (D_g_) of the active enzyme and the distance between crosslinks points (ƹ) as shown in Table 10. At low crosslinking densities, D_g_ is smaller than ƹ and mesh size of the target network is large enough to expose the backbone chain of the polymer (Figure 9A). There is a high probability of the enzyme to cleave the backbone chain of the polymer. This results in a quick digestion of the polymer by the enzyme. However, as the crosslinking density increases, both ƹ and M_c_ of the network decreases. As D_g_ approaches ƹ, the access of enzyme to the backbone chain of the polymer is inhibited (Figure 9B) [62,63] and degradation rate is suppressed. If the crosslinking density is so high that ƹ becomes smaller than D_g_, degradation is stopped hypothetically (Figure 9C). This agrees with the values presented in Table 10. Collagenase (D_g_ = 4.8 nm) is likely to degrade the C0 more quickly than C60 which has a smaller ƹ. The similar trend can be seen for hyaluronidase-assisted degradation of the HA network. In the case of hybrid compositions, S-1 and S-2 show higher degradation rate because of the ƹ values for both are within the range of the D_g_ of collagenase and hyaluronidase. ƹ for S-3 and S-4 is low so the degradation rate for these compositions was even slower.

The average molecular weight between two crosslinks (M_c_) could indicate the extent of mass loss of the network due to degradation. When a scission is made by the enzyme in a network with higher M_c_ value, it could result in a higher mass loss per scission as compared to the network with lower M_c_ value. In Table 10, M_c_ value for C0 is highest and resulted in 100% mass loss in 35 h, whereas C60 with comparatively lower M_c_ value showed only 17% mass loss. Both HA2 and HA20 showed 100% mass loss in 35 h despite their lower M_c_ values as compared to C0 and C60. This is probably due to their hydrophilic character, which dominates the degradation behavior of HA networks. S-1 and S-2 with higher M_c_ values showed higher mass loss of 100% and 60%, respectively as compared to S-3 and S-4 with lower M_c_ values. S-3 and S-4 have similar M_c_ values as HA20; however, they did not show any mass loss. This indicates that hydrophobic character of collagen network controls the degradation behavior of the hybrid scaffolds and hydrophilicity of HA network does not seem to influence their degradation behavior. 

Degradation behavior in physiological fluids can be quite different because of the involved cell activities. In our previous study, we showed that although CDCs were able to attach and proliferate to all hybrid compositions, S-1 and S-2 also experienced some degree of mass loss possibly due to degradation in culture medium (pH 7.4). On the other hand, S-3 and S-4 remained stable during cell culture up to day 7 which also resulted in increase in bending modulus potentially due to ECM deposited by proliferating CDCs. It could be deduced that ECM deposition could also have taken place for S-1 and S-2 by cells, but it could not contribute significantly to bending modulus due to simultaneous mass loss. Therefore, it is desirable to closely control the composition of hybrid scaffolds to achieve the desirable mechanical properties coupled with optimal degradation rate.

## 5. Conclusions

It has been shown that compressive (E*_comp_*) and dynamic moduli (G′, G″) of collagen networks and hybrid scaffolds increase with increasing the crosslinking density under dry as well as wet conditions. However, E*_comp_* in the wet condition could not be measured due to the sensitivity limit of the equipment. An increase in mechanical moduli is attributed to the resistance of the crosslinks towards the polymer chain movement as well as a decrease in the pore size resisting pore edge and wall deformation. The mechanical moduli of collagen/HA hybrids are found to be several orders higher, in both dry and wet conditions, than those of their parent collagen network due to the effect of interlacing between collagen and HA networks. The higher the crosslinking density of the HA network, the greater the increase in the mechanical moduli of collagen/HA hybrid scaffolds. G′ and G″ for all scaffolds decrease, whereas tanδ increases by several orders of magnitude in the wet state due to the lubrication effect of water. Scaffolds with higher crosslinking densities show lower values of water uptake, possibly due to the higher number of water-interacting sites engaged in the crosslinking process. In addition to the effect of crosslinking density, wet mechanical properties of hybrid scaffolds are also found to be affected by the hydrophilicity of the HA network. Dynamic mechanical properties (G′, tanδ) in the wet state matched those of HAVL and exceeded those of HAVL in the dry state. Therefore, it can be concluded that hybrid scaffolds have the potential to serve as mechanically matching TEHV. Furthermore, mechanical properties of an interlaced hybrid scaffold can be optimized for other tissue engineering applications by changing the crosslinking densities of the parent networks.

While mechanical properties decrease significantly in wet conditions, scaffolds could also have a tendency to degrade. The effect of pH, crosslinking density, and enzymatic activity on the degradation behavior of the hybrid scaffolds have been studied. Collagen network and HA network show contrasting behavior with respect to the pH of the degrading medium. This leads to the conclusion that the gross degradation response of collagen/HA hybrid scaffolds can be tuned by varying the collagen: HA network ratio (or choosing the primary network). 

The increase in the concentration of EDC/NHS (or crosslinking density) increased the hydrophobicity of all compositions which delayed the mass loss due to degradation under all conditions. The partial mass loss occurred for hybrid scaffolds in either collagenase or hyaluronidase alone. Higher and accelerated mass loss resulted under the combined effect of collagenase and hyaluronidase. These observations further support the hypothesis that hybrid scaffolds are made up of independent networks of collagen and HA. Controlling the collagen: HA network ratio, as well as the crosslinking density of collagen/HA hybrid scaffolds, provide means to adjust the degradation rates for tissue engineering of organs other than heart valve leaflet. Additionally, pH-sensitive hybrid compositions can be designed for smart biomedical applications. Furthermore, collagen/HA hybrid scaffolds, reported here, with collagen forming the primary network have shown properties similar to fibrosa. Currently, the application of the reported crosslinking approach is under investigation in our lab for the fabrication of hybrid scaffolds with HA and elastin forming primary networks to replicate spongiosa and ventricularis, respectively, to replicate the tri layer structure.

## Figures and Tables

**Table 1:** Nomenclature of parent collagen type I and HA scaffolds.

Sample ID	Conc. of EDC (mM)	Conc. of NHS (mM)
C0	0	0
C25	25	12.5
C40	40	20
C60	60	30
HA2	2	0
HA4	4	0
HA10	10	0
HA20	20	0

**Table 2:** Nomenclature and composition details of collagen/HA hybrid scaffolds.

Sample ID	Primary Collagen Network	Secondary HA Network
S-1	C0	HA2
S-2	C0	HA20
S-3	C60	HA2
S-4	C60	HA20

**Table 3:** Crosslinking densities of collagen network, HA network, and hybrid scaffolds. ^a,b^ represents statistically significant values within collagen networks as compared to C0 and C25, respectively. ^c,d,e^ represent statistically significant values within HA networks as compared to HA2, HA4, and HA10, respectively. ^f,g,h^ represent statistically significant values within hybrid scaffolds as compared to S-1, S-2, and S-3, respectively.

Sample ID	Average Molecular Weight between the Two Crosslinks (M_c_, ×10^2^ kg/mol)	Effective Crosslinking Density (v_e_, kmol/m^3^)	Distance between Crosslinks Points (ƹ, nm)
C0	32 ± 2.5	0.4 ± 0.03	37 ± 1
C25	10 ± 1 ^a^	1.3 ± 0.2	19 ± 1^a^
C40	6.7 ± 2.5 ^a^	2 ± 1 ^a^	15 ± 3 ^a^
C60	4.1 ± 0.5 ^a,b^	3.3 ± 0.4 ^a,b^	11 ± 1 ^a,b^
HA2	6.4 ± 0.9	2 ± 0.3	9.7 ± 1
HA4	5.2 ± 0.2	2.5 ± 0.1	8.3 ± 0.2
HA10	3.3 ± 0.6 ^c^	4.3 ± 0.1	6.0 ± 0.6 ^c,d^
HA20	0.8 ± 0.2 ^c,d,e^	17 ± 4 ^c,d,e^	2.4 ± 0.3 ^c,d,e^
S-1	16 ± 2.1	0.8 ± 0.1	13 ± 1
S-2	14 ± 2.4	0.9 ± 0.1	10 ± 1 ^f^
S-3	7 ± 0.2 ^f,g^	5.0 ± 0.6 ^f,g^	8.2 ± 0.1 ^f^
S-4	5 ± 0.1 ^f,g^	6.8 ± 0.7 ^f,g,h^	7.3 ± 0.1^f,g^

**Table 4:** Compressive modulus (E*_comp_*, kPa) of collagen networks and hybrid scaffolds. ^a,b^ represent statistically significant values (*p* < 0.05) within collagen networks as compared to C0 and C25, respectively. ^c,d,^^e^ represent statistically significant values within hybrid scaffolds as compared to S-1, S-2, and S-3, respectively.

Sample ID	Effective Crosslinking Density (v_e_, kmol/m^3^) [23]	Compression Modulus (E*_comp_*, kPa)
C0	0.4 ± 0.03	35 ± 5
C25	1.3 ± 0.2	56 ± 6 ^a^
C40	2 ± 1 ^a^	63 ± 5 ^a^
C60	3.3 ± 0.4 ^a,b^	68 ± 7 ^a,b^
S-1	0.8 ± 0.1	41 ± 4
S-2	0.9 ± 0.1	73 ± 5 ^c^
S-3	5.0 ± 0.6 ^c,d^	68 ± 5 ^c^
S-4	6.8 ± 0.7 ^c,d,e^	95 ± 4 ^c,d,e^

**Table 5:** Storage modulus G′ (kPa), loss modulus G″ (kPa), and loss tangent (tan δ) of collagen networks and hybrid scaffolds in the dry state (37 °C, 1 Hz). ^a,b,c^ represent statistically significant values within collagen networks as compared to C0, C25, and C40, respectively. ^d,e,f^ represent statistically significant values (*p* < 0.05) within hybrid scaffolds as compared to S-1, S-2, and S-3, respectively.

Sample ID	Effective Crosslinking Density (v_e_, kmol/m^3^)	Rheology Properties		
		Storage Modulus (G′, kPa)	Loss Modulus (G″, kPa)	Loss Tangent (tan δ)
C0	0.4 ± 0.03	16 ± 2	3 ± 1	0.17
C25	1.3 ± 0.2	45 ± 3 ^a^	7 ± 2 ^a^	0.17
C40	2 ± 1 ^a^	49 ± 4 ^a^	7 ± 2 ^a^	0.14
C60	3.3 ± 0.4 ^a,b^	74 ± 3 ^a,b,c^	9 ± 1 ^a^	0.12 ^a,b^
S-1	0.8 ± 0.1	52 ± 6	6 ± 1	0.12
S-2	0.9 ± 0.1	64 ± 4	7 ± 2	0.12
S-3	5.0 ± 0.6 ^d,e^	68 ± 5	7 ± 2	0.11
S-4	6.8 ± 0.7 ^d,e,f^	113 ± 6 ^d,e,f^	10 ± 2 ^d^	0.09 ^d,e^

**Table 6:** Storage modulus G′ (kPa), loss modulus G″ (kPa), and loss tangent (tanδ) of collagen networks and hybrid scaffolds in a hydrated state (37 °C, 1 Hz). ^a,b,c^ represent statistically significant values within collagen networks as compared to C0, C25, and C40, respectively. ^d,e,f^ represent statistically significant values (*p* < 0.05) within hybrid scaffolds as compared to S-1, S-2, and S-3, respectively.

Sample ID	Effective Crosslinking Density (v_e_, kmol/m^3^)	Rheology Properties		
		Storage Modulus (G′, kPa)	Loss Modulus (G″, kPa)	Loss Tangent (tan δ)
>C0	0.4 ± 0.03	>7 ± 2	>9 ± 2	>1.17
C25	>1.3 ± 0.2	10 ± 3	6 ± 1	0.62 ^a^
>C40	2 ± 1 ^a^	>10 ± 3	>5 ± 1	>0.46 ^a^
C60	>3.3 ± 0.4 ^a,b^	18 ± 3 ^a,b,c^	3 ± 1 ^a^	0.18 ^a,b,c^
>S-1	0.8 ± 0.1	>11 ± 3	>4 ± 1	>0.38
S-2	>0.9 ± 0.1	15 ± 3	2 ± 0.6 ^d^	0.24 ^d^
>S-3	5.0 ± 0.6 ^d,e^	>53 ± 4 ^d,e^	>5 ± 2	>0.1 ^d^
S-4	6.8 ± 0.7 ^d,e,f^	55 ± 3 ^d,e^	6 ± 1 ^e^	0.1 ^d^

**Table 7:** Storage modulus G′(Pa), loss modulus G″(Pa), and loss tangent (tanδ) of HA network soaked in PBS at 37 °C. ^a,b,c^ represent statistically significant values (*p* < 0.05) within HA networks as compared to HA2, HA4, and HA10, respectively.

Sample ID	Effective Crosslinking Density (v_e_, kmol/m^3^)	Storage Modulus (G′, Pa)	Loss Modulus (G″, Pa)	Loss Tangent (tanδ)
HA2	2 ± 0.3	320 ± 24	19 ± 6	0.05
HA4	2.5 ± 0.1	480 ± 19 ^a^	19 ± 3	0.04
HA10	4.3 ± 0.1	660 ± 23 ^a,b^	25 ± 6 ^a^	0.04
HA20	17 ± 0.4 ^a,b,c^	870 ± 21 ^a,b,c^	33 ± 4 ^a^	0.03 ^a^

**Table 8:** Comparison of mechanical properties of HAVL and hybrid scaffolds in the dry state.

Mechanical Property	HAVL	Hybrid Scaffolds			
		S-1	S-2	S-3	S-4
Storage Modulus (G′, kPa)	0.545–10.5 kPa(aortic leaflet + sinus) [53]	52 ± 6	64 ± 4	68 ± 5	113 ± 6
Tanδ	0.3–0.4 [53]	0.12	0.12	0.11	0.09

**Table 9:** Comparison of mechanical properties of HAVL and hybrid scaffolds in the wet state.

Mechanical Property	HAVL	Hybrid Scaffolds			
		S-1	S-2	S-3	S-4
Storage modulus (G′, kPa)	0.545–10.5 kPa (aortic leaflet + sinus) [53]	11 ± 3	15 ± 3	53 ± 4	55 ± 3
Tanδ	0.3–0.4 [53]	0.38	0.24	0.1	0.1

**Table 10:** Comparison of the diameter of gyration (D_g_) or molecular weight (M_z_) of the active enzymes with the average molecular weight (M_c_) or average distance between the two crosslinks (ƹ) in the target network. ^a,b^ represents statistically significant values within collagen networks as compared to C0 and C25, respectively. ^c,d,e^ represent statistically significant values within HA networks as compared to HA2, HA4, and HA10, respectively. ^f,g,h^ represent statistically significant values within hybrid scaffolds as compared to S-1, S-2, and S-3, respectively (Please see Table 3).

Active Enzyme with Approx. Molecular Weight (M_z_, kg/mol)	Diameter of Gyration (D_g_, nm)	Target Network	Distance between Crosslinks Points (ƹ, nm)	Average Molecular Weight between the Two Crosslinks (M_c_, ×10^2^ kg/mol)
Collagenase (68–130)	4.8	C0	37 ± 3	32 ± 2.5
		C60	11 ± 1 ^a,b^	4.1 ± 0.5 ^a,b^
Hyaluronidase (55)	4.4	HA2	9.7 ± 1	6.4 ± 0.9
		HA20	2.4 ± 1 ^c,d,e^	0.8 ± 0.2 ^c,d,e^
Collagenase + Hyaluronidase		S-1	13 ± 1	16 ± 2.1
		S-2	10 ± 2	14 ± 2.4
		S-3 ^f,g^	8.2 ± 3 ^f,g^	0.7 ± 0.2 ^f^
		S-4 ^f,g^	7.3 ± 1 ^f,g,h^	0.5 ± 0.1 ^f,g^

## Data Availability

Some of the supporting data is provided in supplementary materials. Data can be provided by corresponding author on request.

## Supplementary Materials

The following are available online at https://www.mdpi.com/2079-4983/12/1/20/s1, Figure S1: Scanning electron images of (A) C60 as primary collagen network, (B) HA20 as secondary HA network, and (C) S-4 as the result-ant hybrid scaffold at 200×, 10 kV.

## References

[1] Gandaglia A., Bagno A., Naso F., Spina M., Gerosa G. (2011). Cells, scaffolds and bioreactors for tissue-engineered heart valves: A journey from basic concepts to contemporary developmental innovations. Eur. J. Cardiothorac. Surg..

[2] Ward B.B., E Brown S., Krebsbach P.H. (2010). Bioengineering strategies for regeneration of craniofacial bone: A review of emerging technologies. Oral Dis..

[3] Walmsley G.G., Cheung A.T., Hu M.S., Lorenz H.P., Longaker M.T. (2016). Osteogenic Differentiation of Adipose-Derived Stromal Cells: Advancements and Future Directions for Bone Tissue Engineering. Sci. Proc..

[4] O’Brien F.J. (2011). Biomaterials & scaffolds for tissue engineering. Mater. Today.

[5] Hollister S., Maddox R., Taboas J. (2002). Optimal design and fabrication of scaffolds to mimic tissue properties and satisfy biological constraints. Biomaterials.

[6] Hutmacher D.W. (2000). Scaffolds in tissue engineering bone and cartilage. Biomaterials.

[7] Drury J.L., Mooney D.J. (2003). Hydrogels for tissue engineering: Scaffold design variables and applications. Biomaterials.

[8] Lanza R., Langer R., Vacanti J.P. (2011). Principles of Tissue Engineering. Elsevier Sci..

[9] Meyer U., Meyer T., Handschel J., Wiesmann H.P. (2009). Fundamentals of Tissue Engineering and Regenerative Medicine.

[10] Henkel J., Woodruff M.A., Epari D.R., Steck R., Glatt V., Dickinson I.C., Choong P.F.M., Schuetz M.A., Hutmacher D.W. (2013). Bone Regeneration Based on Tissue Engineering Conceptions: A 21st Century Perspective. Bone. Res..

[11] Veselý I. (2006). Heart Valve Tissue Engineering. Wiley Encycl. Biomed. Eng..

[12] Zhang L., Hu J., Athanasiou K.A. (2009). The Role of Tissue Engineering in Articular Cartilage Repair and Regeneration. Crit. Rev. Biomed. Eng..

[13] Azevedo H., Reis R.L. (2004). Understanding the Enzymatic Degradation of Biodegradable Polymers and Strategies to Control Their Degradation Rate.

[14] Blackwood K.A., Bock N., Dargaville T.R., Woodruff M.A. (2012). Scaffolds for Growth Factor Delivery as Applied to Bone Tissue Engineering. Int. J. Polym. Sci..

[15] Garg T., Singh O., Arora S., Murthy R.S.R. (2012). Scaffold: A Novel Carrier for Cell and Drug Delivery. Crit. Rev. Ther. Drug Carr. Syst..

[16] Driessen N.J., Mol A., Bouten C.V., Baaijens F.P. (2007). Modeling the mechanics of tissue-engineered human heart valve leaflets. J. Biomech..

[17] Hasan A., Ragaert K., Swieszkowski W., Selimović Š., Paul A., Camci-Unal G., Mofrad M., Khademhosseini A. (2014). Biomechanical properties of native and tissue engineered heart valve constructs. J. Biomech..

[18] Hilbert S.L., Schöen F.J., Ferrans V.J. (2005). Biomechanics: Allograft Heart Valves. Cardiac Reconstructions with Allograft Tissues.

[19] Sacks M.S., Merryman W.D., Schmidt D.E. (2009). On the biomechanics of heart valve function. J. Biomech..

[20] Eckert C.E., Fan R., Mikulis B., Barron M., Carruthers C.A., Friebe V.M., Vyavahare N.R., Sacks M.S. (2013). On the biomechanical role of glycosaminoglycans in the aortic heart valve leaflet. Acta. Biomater..

[21] Dabiri Y., Ronsky J., Ali I., Basha A., Bhanji A., Narine K. (2016). Effects of Leaflet Design on Transvalvular Gradients of Bioprosthetic Heart Valves. Cardiovasc. Eng. Technol..

[22] Gould P.L., Cataloglu A., Clark R.E. (1976). Mathematical modelling of human aortic valve leaflets. Appl. Math. Model..

[23] Nazir R., Bruyneel A., Carr C., Czernuszka J. (2019). Collagen type I and hyaluronic acid based hybrid scaffolds for heart valve tissue engineering. Biopolymers.

[24] Ma L., Gao C., Mao Z., Zhou J., Shen J., Hu X., Han C. (2003). Collagen/chitosan porous scaffolds with improved biostability for skin tissue engineering. Biomaterials.

[25] Ma J., Wang H., He B., Chen J. (2001). A preliminary in vitro study on the fabrication and tissue engineering applications of a novel chitosan bilayer material as a scaffold of human neofetal dermal fibroblasts. Biomaterials.

[26] Dhandayuthapani B., Yoshida Y., Maekawa T., Kumar D.S. (2011). Polymeric Scaffolds in Tissue Engineering Application: A Review. Int. J. Polym. Sci..

[27] Engineer C., Parikh J., Raval A. (2011). Review on hydrolytic degradation behavior of biodegradable polymers from controlled drug delivery system. Trends. Biomater. Artif. Organs.

[28] Yildirimer L., Buanz A., Gaisford S., Malins E.L., Becer C.R., Moiemen N., Reynolds G., Seifalian A.M. (2015). Controllable degradation kinetics of POSS nanoparti-cle-integrated poly (ε-caprolactone urea) urethane elastomers for tissue engineering applications. Sci. Rep..

[29] Gueye P., Splinter R. (2011). Handbook of Physics in Medicine and Biology. Med. Phys..

[30] Ducheyne P., Healy K., Hutmacher D.E., Grainger D.W., Kirkpatrick C.J. (2011). Comprehensive Biomaterials. Elsevier Sci..

[31] Gad S.C., Gad-McDonald S. (2015). Biomaterials, Medical Devices, and Combination Products: Biocompatibility Testing and Safety Assessment.

[32] Adeosun S.O., Lawal G.I., Gbenebor O.P. (2014). Characteristics of Biodegradable Implants. J. Miner. Mater. Charact. Eng..

[33] Neves N., Mano J.F., Gomes M.E., Marques A.P., Azevedo H.S. (2008). Natural-Based Polymers for Biomedical Applications.

[34] Gerweck L.E., Seetharaman K. (1996). Cellular pH gradient in tumor versus normal tissue: Potential exploitation for the treatment of cancer. Cancer Res..

[35] Tannock I.F., Rotin D. (1989). Acid pH in tumors and its potential for therapeutic exploitation. Cancer Res..

[36] Acharya U.R., Joseph K.P., Kannathal N., Lim C.M., Suri J.S. (2006). Heart rate variability: A review. Med. Biol. Eng. Comput..

[37] Chen Q., Bruyneel A., Clarke K., Carr C., Czernuszka J. (2012). Collagen-based Scaffolds for potential application of heart valve tissue engineering. J. Tissue Sci. Eng..

[38] Loh Q.L., Choong C. (2013). Three-Dimensional Scaffolds for Tissue Engineering Applications: Role of Porosity and Pore Size. Tissue Eng. Part B Rev..

[39] Shonaike G.O., Advani S.G. (2003). Advanced Polymeric Materials: Structure Property Relationships.

[40] Fernández F.I., Moakes R.J.A. (2014). Norton IT. Designing biopolymer fluid gels: A microstructural approach. Food Hydrocol..

[41] Vincent R.R.R., Mansel B.W., Kramer A., Kroy K., Williams M.A.K. (2013). Micro-rheological behaviour and nonlinear rheology of networks assembled from polysaccharides from the plant cell wall. New J. Phys..

[42] Urdl K., Kandelbauer A., Kern W., Müller U., Thebault M., Zikulnig-Rusch E. (2017). Self-healing of densely crosslinked thermoset polymers: A critical review. Prog. Org. Coat..

[43] Papakonstantopoulos G.J., Feher F.J., Alwardt C.L., Lee B.J. (2017). Rubber Composition Having a Crosslink Distribution, Its Preparation and Article with Component.

[44] Davidenko N., Schuster C., Bax D., Raynal N., Farndale R., Best S., Cameron R. (2015). Control of crosslinking for tailoring collagen-based scaffolds stability and mechanics. Acta. Biomater..

[45] Baier L.J., Bivens K.A., Patrick C.W., Schmidt C.E. (2003). Photocrosslinked hyaluronic acid hydrogels: Natural, biodegradable tissue engineering scaffolds. Biotech. Bioeng..

[46] Lam J., Truong N.F., Segura T. (2014). Design of cell–matrix interactions in hyaluronic acid hydrogel scaffolds. Acta. Biomater..

[47] Zustiak S.P., Wei Y., Leach J.B. (2013). Protein–Hydrogel Interactions in Tissue Engineering: Mechanisms and Applications. Tissue Eng. Part B Rev..

[48] Ebnesajjad S. (2012). Handbook of Biopolymers and Biodegradable Plastics: Properties, Processing and Applications.

[49] Sewell-Loftin M.K., Chun Y.W., Khademhosseini A., Merryman W.D. (2011). EMT-Inducing Biomaterials for Heart Valve Engineering: Taking Cues from Developmental Biology. J. Cardiovasc. Transl. Res..

[50] Mavrilas D., Missirlis Y. (1991). An approach to the optimization of preparation of bioprosthetic heart valves. J. Biomech..

[51] Merryman W.D., Huang H.Y.S., Schoen F.J., Sacks M.S. (2006). The effects of cellular contraction on aortic valve leaflet flexural stiffness. J. Biomech..

[52] Puperi D.S., O’Connell R.W., Punske Z.E., Wu Y., West J.L., Grande-Allen K.J. (2016). Hyaluronan Hydrogels for a Biomimetic Spongiosa Layer of Tissue Engineered Heart Valve Scaffolds. Biomacromolecules.

[53] Jiao T., Clifton R.J., Converse G.L., Hopkins R.A. (2012). Measurements of the Effects of Decellularization on Viscoelastic Properties of Tissues in Ovine, Baboon, and Human Heart Valves. Tissue Eng. Part A.

[54] Nazir R. (2016). Collagen–hyaluronic acid based interpenetrating polymer networks as tissue engineered heart valve. Mater. Sci. Technol..

[55] Chapman C.L. (2007). Engineering TUoTaAB. Gas Permeation through Nanoporous Polycarbonate Track-Etched Membranes: Pulsed Plasma Polymerization of Thin Coatings to Modulate Gas Permeability.

[56] Testa B., Mayer J.M. (2003). Hydrolysis in Drug and Prodrug Metabolism.

[57] Compton R.G., Bamford C.H., Tipper C.F.H. (1972). Ester Formation and Hydrolysis and Related Reactions. Elsevier Sci..

[58] Patterson J., Siew R., Herring S.W., Lin A.S., Guldberg R., Stayton P.S. (2010). Hyaluronic acid hydrogels with controlled degradation properties for oriented bone regeneration. Biomaterials.

[59] Mojarradi H. (2010). Coupling of Substances containing a primary Amine to Hyaluronan via Carbodiimide-mediated Amidation.

[60] Hoffman A.S. (2012). Hydrogels for biomedical applications. Adv. Drug Deliv. Rev..

[61] Zhong S., Campoccia D., Doherty P., Williams R., Benedetti L., Williams D. (1994). Biodegradation of hyaluronic acid derivatives by hyaluronidase. Biomaterials.

[62] Vasile C. (2009). Environmentally Degradable Materials based on Multicomponent Polymeric Systems.

[63] Salamone J.C. (1996). Polymeric Materials Encyclopedia.

