# Supplementary material for: Mechanical and Degradation Properties of Hybrid Scaffolds for Tissue Engineered Heart Valve (TEHV)
Source: J Funct Biomater. 2021 Mar 9;12(1):20. doi: 10.3390/jfb12010020 (PMC8006234; doi:10.3390/jfb12010020)
Supplement: Supplementary file 1 [file jfb-12-00020-s001.pdf]

Journal of
Functional Biomaterials

Article

Mechanical and Degradation Properties of Hybrid Scaffolds for Tissue Engineered Heart Valve (TEHV)

Rabia Nazir [1,2,*], Arne Bruyneel [3], Carolyn Carr [3] and Jan Czernuszka [1]

[1] Department of Materials, University of Oxford, Parks Road, Oxford OX1 3PH, UK; jan.czernuszka@materials.ox.ac.uk
[2] Interdisciplinary Research Centre in Biomedical Materials (IRCBM), COMSATS University Islamabad (CUI), Lahore Campus, Lahore 54000, Pakistan
[3] Department of Physiology, Anatomy and Genetics, University of Oxford, Parks Road, Oxford OX1 3PT, UK; arne.bruyneel@gmail.com (A.B.); carolyn.carr@dpag.ox.ac.uk (C.C.)
* Correspondence: author: rabia_516@yahoo.com

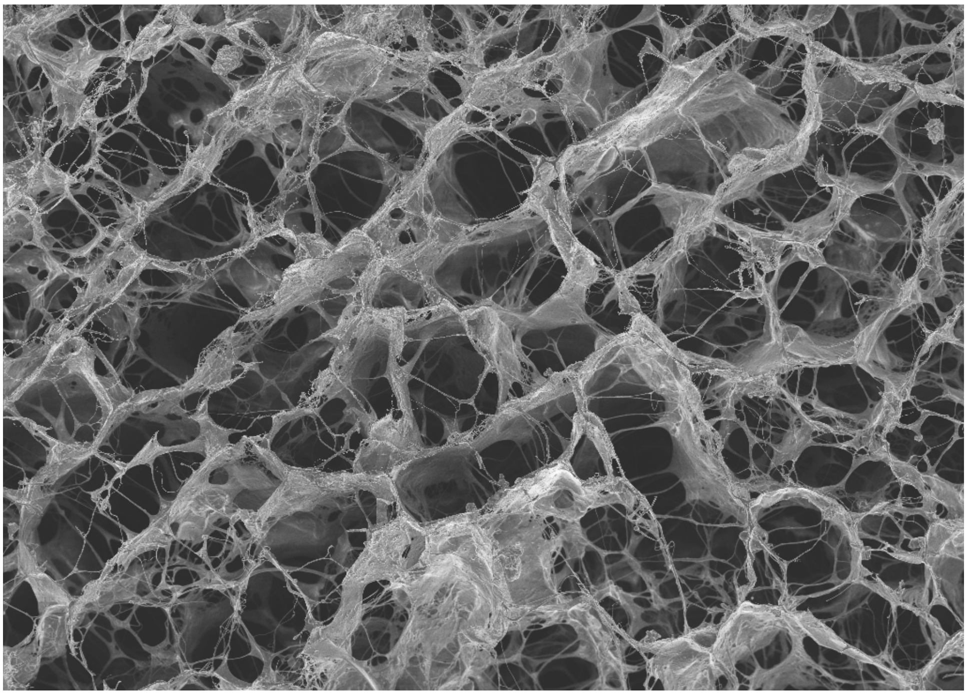

(A)

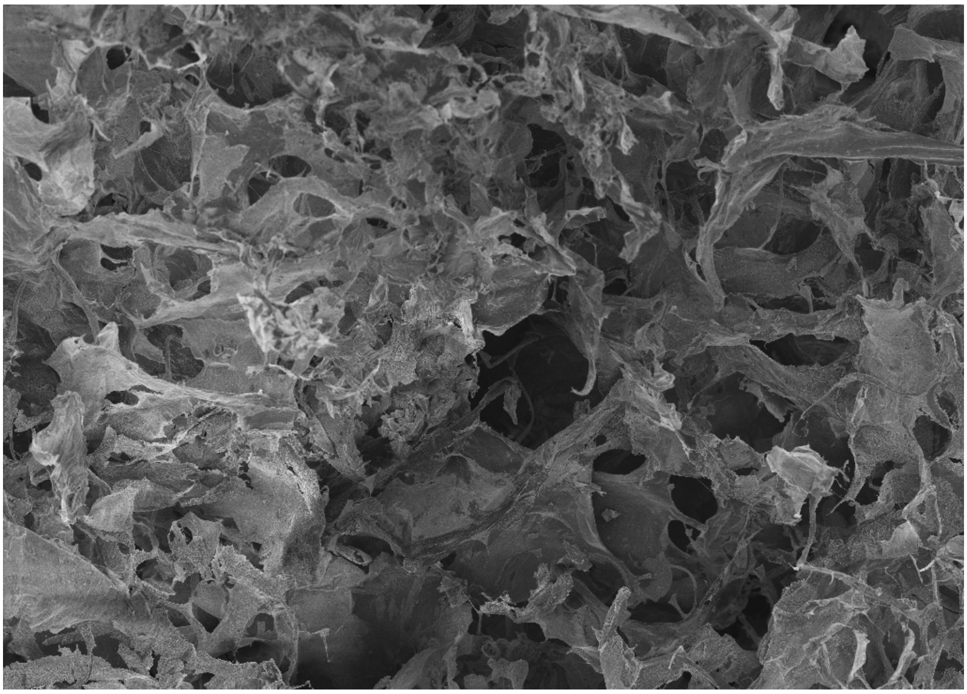

(**B**)

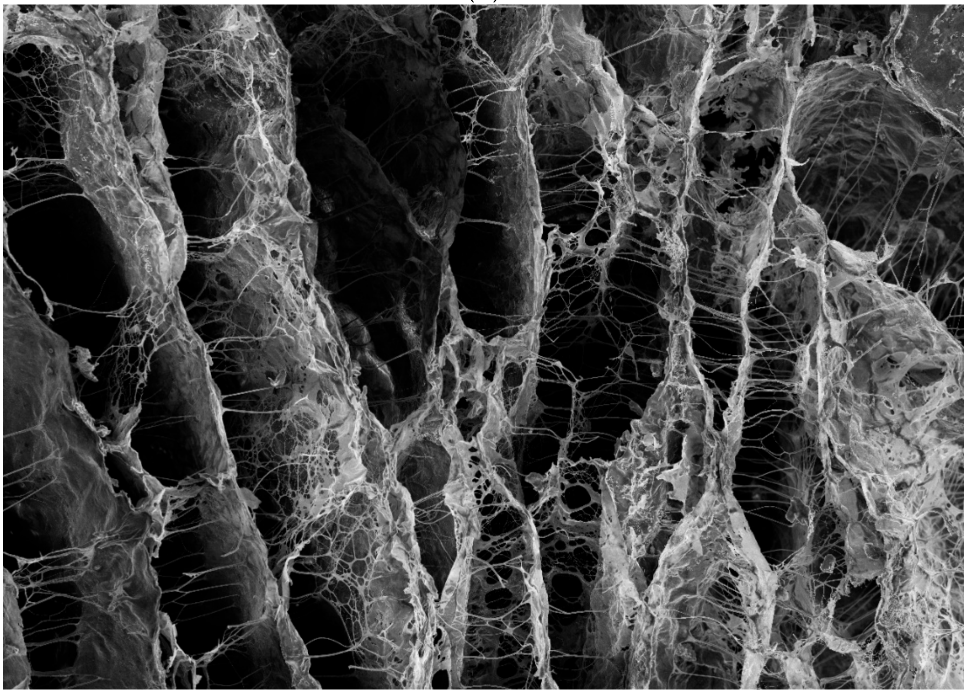

(**C**)

Figure S1. Scanning electron images of (**A**) C60 as primary collagen network, (**B**) HA20 as secondary HA network, and (**C**) S-4 as the resultant hybrid scaffold at 200×, 10kV.

Scanning electron microscope (SEM, JEOL JSM-840F) was employed for imaging of surfaces using secondary electron imaging. Samples were sectioned with a sharp razor blade and mounted onto 12 mm diameter aluminum stubs using adhesive carbon tabs. All samples were coated with 3.0 nm platinum coating using a 208HR sputter coater (Cressington Scientific Instruments Ltd, UK). SEM images of scaffolds (five images/sample) were taken at an accelerating voltage of 10 kV.
